# Health-Related Quality of Life in Adult Spanish Women with Endometriomas or Deep Infiltrating Endometriosis: A Case-Control Study

**DOI:** 10.3390/ijerph18115586

**Published:** 2021-05-24

**Authors:** Evdochia Adoamnei, Inés Morán-Sánchez, María Luisa Sánchez-Ferrer, Jaime Mendiola, María Teresa Prieto-Sánchez, Miriam Moñino-García, Joaquín A. Palomar-Rodríguez, Alberto Manuel Torres-Cantero

**Affiliations:** 1 Division of Preventive Medicine and Public Health, Department of Public Health Sciences, University of Murcia School of Medicine, Espinardo, 30100 Murcia, Spain; evdochia.adoamnei@um.es (E.A.); jaime.mendiola@um.es (J.M.); miriam.monino@carm.es (M.M.-G.); amtorres@um.es (A.M.T.-C.); 2 Institute for Biomedical Research of Murcia, IMIB-Arrixaca, El Palmar, 30120 Murcia, Spain; ines.moran@carm.es (I.M.-S.); mt.prieto@um.es (M.T.P.-S.); 3 Mental Health Centre, Cartagena, 30201 Murcia, Spain; 4 Department of Obstetrics & Gynecology, “Virgen de la Arrixaca” University Clinical Hospital, El Palmar, 30120 Murcia, Spain; 5 Biomedical Research Centre Network for Epidemiology and Public Health (CIBERESP), 28029 Madrid, Spain; 6 Servicio de Planificación y Financiación Sanitaria, Consejería de Salud, Región de Murcia, 30001 Murcia, Spain; joaquin.palomar@carm.es

**Keywords:** case-control study, endometriosis, health-related quality of life, SF-12v2

## Abstract

Endometriosis is a disabling disease that may significantly compromise a woman’s social relationships, sexuality, and mental health. Considering the impact of endometriomas and deep infiltrating endometriosis (DIE) on quality of life and the limited number of papers on this topic, the objective of this study was to assess health-related quality of life (HRQoL) in adult Spanish women with the condition. A case-control study was conducted on a group of 99 patients with ovarian endometriomas or DIE and 157 controls. Women underwent physical and gynecological examinations, and they completed health questionnaires including the Short Form-12v2 (SF-12v2), a survey for HRQoL. Eight scales and two component summary scores (Physical (PCS) and Mental (MCS), respectively) were calculated. Women with endometriomas or DIE had significantly worse PCS: 47.7 ± 9.7 vs. 56.1 ± 5.9, respectively (*p* < 0.001) compared to controls, as well as lower scores on seven out of the eight scales (*p* < 0.01). No significant differences were found for the MCS. Conclusions: HRQoL was significantly lower in patients with endometriomas or DIE compared to controls. If confirmed, these results may have important implications for prevention, clinical practice, and intervention.

## 1. Introduction

Endometriosis is defined as a benign and proliferative chronic disease characterized by endometrial glands and stroma outside the uterus, frequently in the peritoneal spaces [1]. A prevalence of about 10% has been estimated, affecting between 4% and 30% of women during childbearing age [2]. The most frequently reported effects in patients with endometriosis are chronic pelvic pain, dysmenorrhea, dyschezia, dyspareunia, and infertility [3]. Cystic ovarian endometriosis or endometriomas and deeply infiltrating endometriosis (DIE) are the two most important manifestations of endometriosis [1]. DIE occurs when endometriotic pelvic implants more than 5 mm under the peritoneal surface in the parametria, Douglas pouch, anterior rectal wall, posterior vaginal fornix, antero-uterine pouch, bladder detrusor, ureters, or sigmoid colon [1,4]. Clinically, endometriomas are reportedly the most common, accounting for 17–44% of patients with endometriosis [5] Despite its lower prevalence, DIE is consistently known to be strongly associated with severe pain or sexual dysfunction [6]. For these reasons, endometriosis is considered a disabling disorder that may significantly compromise mental health, social relationships, and sexuality [7]. Therefore, this condition can negatively affect a woman’s quality of life [8]. Several hypotheses regarding the pathogenesis of endometriosis have been suggested, including its potential prenatal origin or the exposure to environmental endocrine disruptors as a potential risk factor for the development of the disease [9,10,11].

In recent years, there has been growing interest in incorporating the assessment of health-related quality of life (HRQoL) in the clinical studies and routine clinical management of diseases, including endometriosis. Several studies have addressed the association between quality of life and endometriosis [12,13,14,15,16,17,18,19,20,21,22]. However, in general, those studies have been limited by small sample sizes, heterogeneities between study populations, and evaluating tools, while the impact of confounders such as age, income, and symptom severity have been insufficiently explored. Moreover, the majority of the studies have focused on series of cases, the evaluation of randomized clinical trials (with surgery or drugs), or pre- and post-operative changes in quality of life of women with endometriosis [12,23].

To the best of our knowledge, no previous study has evaluated the relationships between HRQoL and presence of endometriomas or DIE after controlling for confounding factors in adult Spanish women. We sought to assess whether endometriomas or DIE have a significant impact on HRQoL in adult women. We hypothesized that women with endometriosis would have worse HRQoL scores than controls.

## 2. Materials and Methods

### 2.1. Study Design and Participants

We conducted a case-control study between September 2014 and May 2015 in a tertiary hospital from Murcia Region (Spain). Cases (*n* = 99) were women attending the hospital’s Endometriosis Unit. Endometriosis diagnoses were established by clinical history and confirmed by transvaginal ultrasound (TVUS) as having endometriomas (*n* = 79) or DIE (*n* = 20) [1,24,25]. Controls were women without the disease who attended the gynecological outpatient clinics for regular examinations (*n* = 157). Women who were pregnant, undergoing oncological treatment, or had genitourinary prolapse were excluded from the study. Women attending the outpatient clinic at the hospital were recruited for the study by gynecologists. More than 95% of them agreed to participate and signing a informed consent form, with refusal mostly linked to a lack of time to complete the questionnaires. This study was approved by the Ethics Review Board of the University of Murcia on 3 October 2013. Moreover, this article follows the STROBE guidelines for reporting observational studies [26].

### 2.2. Gynecological History and Physical Examination

Women completed health questionnaires, were asked about gynecological history, and underwent a gynecological examination including TVUS (Voluson E-8^®^ and 4–9 MHz transducer, General Electric Healthcare, Fairfield, CT, USA) at a scheduled clinical visit. For ultrasound examinations, the methods described by Abrao et al. for DIE cases [24] and Guerriero et al. for ovarian endometriomas cases [25] were followed. Women with both types of endometriosis were classified as DIE. All women with endometriosis were diagnosed by 2D ultrasound, except for DIE (20% of cases), in which case 3D ultrasound was also used. A visual analog scale (0–10) was employed to assess endometriosis-associated pelvic pain at the time of the examination [27]. Height and weight were measured using a digital scale (Tanita SC 330-S, Amsterdam, The Netherlands). All clinical evaluations were performed by two gynecologists using the same methodology.

### 2.3. Health-Related Quality of Life Measurement

The Short Form (SF)-12v2 Health Survey is a validated short version of the SF-36v2 generic questionnaire that encompasses 12 items evaluating physical and mental health from the participant’s point of view (four weeks recall period) [28,29,30]. The questionnaire generates eight scales: general health, physical functioning, physical role, emotional role; bodily pain, mental health, vitality, and social functioning. All raw scale scores were converted to a 0–100 scale, with higher scores representing higher levels of HRQoL. The scales were also transformed to normative-based scores according to the SF-12v2 recommendations to give a mean of 50 and a standard deviation (SD) of 10 using a representative sample of the general population of the USA in 1998 [28,29,30]. This transformation allowed us to obtain two summary measures, the Physical and Mental Component Summary (PCS and MCS, respectively), that could be directly compared to other scales and scores. As the mean score was set to 50, scores higher than 50 or lower than 50 indicated better or worse physical or mental health, respectively, than the 1998 USA general population. Score bounds were set at 48 ± 0.2 for a small effect on HRQoL, 45 ± 0.5 for a moderate effect on HRQoL, and ≤42 ± 0.8 for a large effect on HRQoL [16,17,31,32]. Overall, the SF-12v2 was a valid tool to assess HRQoL in our setting and has been previously used in Spanish populations [28,30,33].

### 2.4. Statistical Analyses

We reported categorical variables as frequencies, and we reported valid percentages and continuous variables as mean/median and standard deviations. Group differences in continuous variables were analyzed using unpaired Student’s *t* tests, and differences between groups on categorical variables were compared with Pearson’s χ^2^. An analysis of covariance was used to calculate adjusted crude (0–100) and norm-based scales, as well as component summary differences between cases and controls, while taking relevant covariates into account. A multiple logistic regression model was used to explore associations between cases and controls, as well as norm-based scales and summary measure score with a cut-off of above/below 50 using odds ratios (ORs) and 95% CI. The model was adjusted for the following potential sources of confounding: age, educational level, marital status, infertility problems, and current occupation. based on previous publications, we decided that it would be adequate to detect a difference of at least 3 points (with an SD of about 7 points) in the global scores (PCS or MCS) between cases and controls for sample size calculation. If we accepted a risk of 0.05 and a statistical power of 80% to detect differences, if there were any, we would have needed 90 women in each group for a total of 180. All tests were two-tailed with a 0.05 significance level. All analyses were conducted using IBM SPSS 25.0 (IBM, New York, NY, USA).

## 3. Results

### 3.1. Comparison of the General Characteristics of Cases of Endometriosis and Controls

For the entire population, controls were younger and showed a higher educational level than cases, and there were also differences regarding marital status and current occupation (Table 1).

### 3.2. Comparison of the Health Concept Scales of the SF-12v2 Questionnaire between Cases and Controls

Table 2 shows comparisons of the health concept scales of the SF-12v2 questionnaire between cases and controls. The crude and adjusted analyses presented similar results, reporting significantly lower scores for cases in seven of the eight scales. There were no statistically significant differences in mental health scales scores between the groups.

### 3.3. Comparison of the Norm-Based Scales and Summary Measure Scores of SF-12v2 between Cases and Controls

**Figure 1:**
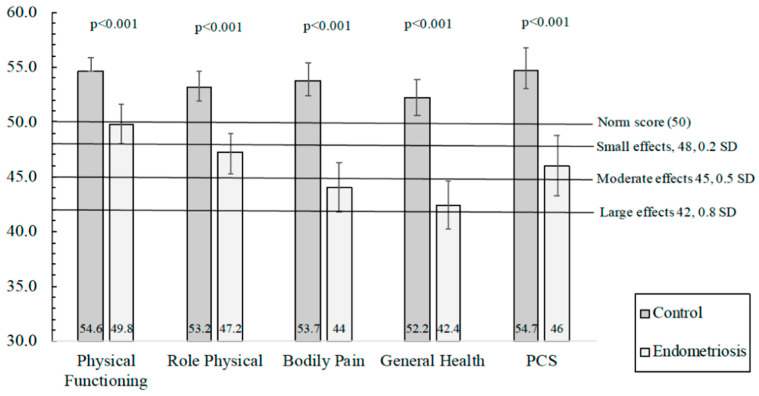
Adjusted means and 95% CI of the four scales and the Physical Component Summary (PCS) of the SF-12v2 questionnaire. The model was adjusted by age, educational level, marital status, infertility problems, and current occupation (ANCOVA).

**Figure 2:**
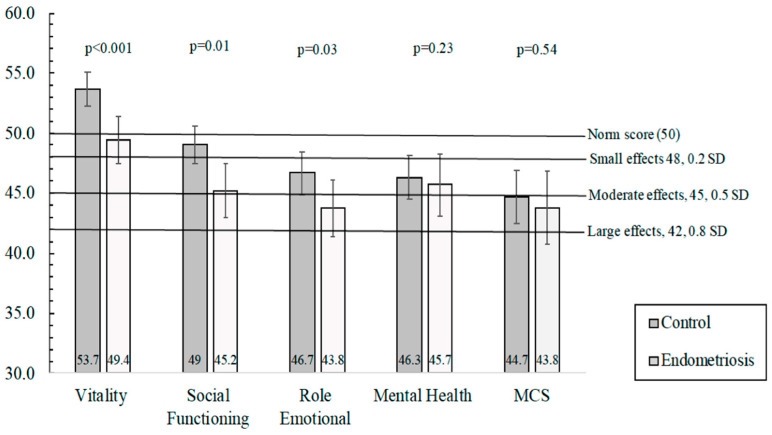
Adjusted means and 95% CI of the four scales and Mental Component Summary (MCS) of the SF-12v2 questionnaire. Model adjusted by age, educational level, marital status, infertility problems and current occupation (ANCOVA).

A comparison of the norm-based scales and summary measure scores of the SF-12v2 between cases and controls is presented in Table 3. The four scales (physical functioning, physical role, bodily pain, and general health) and the PCS showed significantly lower values for cases, and equivalent results were observed in adjusted analyses. Figure 1 shows the adjusted means and 95% CI of the four scales and PCS. Regarding the four scales (vitality, social functioning, emotional role, and mental health) and the MCS, significantly lower scores for vitality, social functioning, and emotional role were detected for cases (Table 3), and comparable results were detected in adjusted models. Figure 2 shows the adjusted means and 95% CI of the four scales and MCS. However, the MCS was similar between both groups.

The crude and adjusted OR and the 95% CI for the norm-based scales and summary measure scores of SF-12v2 between cases and controls were also calculated while taking a cut-off of 50 to indicate better or worse mental or physical health than the mean US population. Women with endometriosis were 8.36 times (95% CI: 4.06–17.2) more likely than controls to have PCS scores <50. The strength of this relationship remained significant when the analysis was conducted for the PCS subscales (physical functioning, physical role, bodily pain, and general health), with ORs ranging between 3.4 and 6.5 (*p* < 0.05). For example, women with endometriosis (compared to controls) were 2.65 (1.48–4.75) or 2.33 (1.29–4.22) times more likely to have vitality or social functioning scores <50, respectively. However, when considering the MCS and its scales, only vitality, social functioning, and mental health remained significantly associated after multivariate adjustment. Nevertheless, no significant crude or adjusted associations were found with the MCS. For women with only endometriomas (*n* = 79), a sensitivity analysis was conducted, and it showed similar results compared to the total cases.

The mean score was set to 50. Scores ≥ 50 or <50 indicate better or worse, respectively, physical or mental health than the mean norm-based scores in the US population.

## 4. Discussion

The HRQoL was lower in the large majority of the health scales (seven out of eight) and the PCS of the SF-12v2 questionnaire in patients with endometriosis compared to controls. This fact suggests that endometriosis may play an important role in these women’s quality of life. Moreover, to our knowledge, no previous study has assessed associations between the HRQoL and presence of endometriomas or DIE in adult Spanish women.

Several studies have evaluated HRQoL in women with endometriosis, and the presence of an impaired quality of life in this condition has been established. However, most of these studies assessed changes in HRQoL after randomized clinical trials—with surgery or drugs—or pre- and post-operative interventions in women with endometriomas or DIE [12,23,34,35,36]. Therefore, there is a lack of information regarding analytic observational studies that have considered associations between HRQoL and the presence or absence of endometriosis. These studies could shed light on the occurrence, magnitude, prevention, and potential interventions associated with HRQoL in endometriosis. Perceived HRQoL in patients with endometriosis is most commonly associated with illness acceptance, dyspareunia, BMI, and the negative impact of symptoms on the relationship with the partner [37].

Many validated questionaries, such as the WHOQOL-BREF quality of life questionnaire [37] and the WERFEPHect Clinical Questionnaire, have been used to asses HRQoL [38]. There is a specific Endometriosis Health Profile-30 (EHP-30) questionnaire [39] that was recently validated in Spanish [40]. Only a few observational studies have explored HRQoL in endometriosis using the SF-36 or SF-12 questionnaires [12,41,42]. In a case-control study on 103 German women, Siedentopf and colleagues reported that HRQoL, for both PCS and MCS, was significantly reduced in endometriosis [21]. Comparable results were obtained for Italian women that had endometriosis with pelvic pain [19]. Fourquet and collaborators studied HRQoL in 193 Puerto Rican women with endometriosis and showed that PCS and MCS were relatively low (means of 38.4 and 39.5, respectively) [18], indicating substantial disability (≤40 points) [43]. Similar results were detected in Swedish women with endometriosis, reporting significantly lower HRQoL scores than the general female population [20]. In an international cross-sectional survey (*n* = 931), HRQoL was significantly reduced in all eight scores, as well as both summary components, compared to norm-based scores from the general US population [17]. The same research group showed that Dutch women with endometriosis reported a greater impairment of HRQoL in both the mental and physical scales compared to a control group [16]. Recently, in a case-control study, US adolescents with endometriosis presented significantly poorer summary components (PCS and MCS) and eight-scale scores [22]. In a cross-sectional study, Pessoa et al. [41] reported that median scores in the HRQoL domains (physical functioning, emotional role, and general health), and clinical symptoms were significantly associated among different types of dyspareunia. Regarding pain outside of menstruation, there was significance in the pain domain, and the degree of pain was significantly associated with the physical functioning and physical role domains. Interestingly enough, it was not the stage of endometriosis that interfered in the HRQoL of women with endometriosis and infertility; rather, it was the clinical manifestations, such as pain or dyspareunia.

Our results were moderately consistent with previous studies that showed similar findings. In our study, HRQoL was diminished in seven of the eight health scales and the PCS in cases compared to controls. Moreover, women with endometriosis presented values of around 45 for some of the scales (physical role and bodily pain), indicating a moderate influence of the disease on those aspects of HRQoL. Likewise, cases also showed values of around 45 for a few scales of the MCS (social functioning and mental health). It is worth mentioning that the general health and emotional role scores were about 42, denoting a potentially large effect on HRQoL [15,32].

In our current study, MCS scores were relatively low (<50), mainly for cases (mean = 43.8). However, no significant differences between cases and controls were observed. In this regard, our results were not consistent with most of the previous studies [16,17,21,22]. These divergent findings could have been due to dissimilarities in study populations or disease severity. For instance, it is important consider that the participants of our study were enrolled in a tertiary hospital, so results may vary from other types of populations [15]. Moreover, no significant differences in HRQoL between patients with asymptomatic endometriosis and controls have been shown, indicating that endometriosis symptoms or severity may be important in this regard [19,44]. In our study, only 20 women presented DIE, a more severe form of the disease, but given the relatively small sample, a sensitivity analysis would not provide meaningful results in terms of global interpretation. For the 79 women with endometriomas, similar results were obtained.

On the other hand, longitudinal studies would be necessary to confirm the potential causal assumptions reported here and to allow for the assessment of the effects of disease progression and related symptoms on women’s HRQoL. Endometriosis is a chronic disease that, in most patients, can only be “controlled” [1]. A treatment with the sole objective of eradicating the subjacent disease is not enough. To improve the HRQoL of these patients, medical care must also address the sexual, social, and emotional problems associated with endometriosis [17]. Modifiable factors like diet and physical activity should also be evaluated as possible targets for enhancing HRQoL in these women. Additionally, multiple and pervasive effects are expected to materially alter the life-course trajectory of women with the disease. Unfortunately, current practice models too often result in a prolonged delay between the symptom onset, diagnosis, and treatment of endometriosis, increasing the impact on life-course [45]. Missmer et al. [45] identified many intrinsic and extrinsic characteristics (e.g., fatigue and age) that can modulate the impact of endometriosis on life domains. Given the importance of the early diagnosis of endometriosis, gynecologist should not only ask adolescent patients about the prevention of sexually transmitted infections and contraception but also inquire, for instance, about dyspareunia. Three quarters of adolescents with endometriosis were found to have experienced dyspareunia that had a significant and negative impact on their HRQoL and may impact physical and mental well-being [46]. Moreover, Warzecha et al. [38] reported that the prevalence of depression was positively correlated with the onset of dyspareunia. Chronic pelvic pain and painful defecation increase the risk of depressive disorder. Endometriosis-related symptoms and pain severity were found to correlate with the prevalence of depression. Additionally, the stage of endometriosis has been significantly related to the prevalence of infertility [38]. The lower HRQoL found among women with endometriosis versus those without the disease during in vitro fertilization (IVF) treatment highlights the importance of developing strategies to improve their HRQoL [47]). Lasty, self-efficacy is also significantly associated with both the mental and physical quality of life. A patient´s perception of their ability to manage pain and how much uncertainty they feel about their endometriosis are important factors for HRQoL, especially in the physical domain [48]. Supporting patients with endometriosis to improve self-efficacy through a structured chronic disease management program may lead to improvements in this aspect of their wellbeing [42].

On the basis of our findings, we may recommend a multidisciplinary approach to endometriosis care [10]. A patient-centered approach, with broad cooperation between disciplines, such as sexologists, psychotherapists, and pain specialists, could be a valuable strategy for addressing these challenges and improving the long-term care of patients with endometriosis [17]. Endometriosis substantially affects overall well-being, and psychological interventions leading to restructure coping styles and receive psychosocial support may improve HRQoL [14]. To our knowledge, this is the first report exploring this matter in adult Spanish women.

Our study was subject to limitations that we would be remiss not to point out. In case-control designs, selection and measurement bias must always be taken into account. Nevertheless, controls were women attending the same hospital in the same period who were from the same population from which the endometriosis cases came. The misclassification of disease status may have occurred, but, if present, it would have contributed to an underestimation of the true magnitude of associations.

## 5. Conclusions

In conclusion, our findings support the hypothesis that HRQoL is significantly impaired in adult Spanish patients with endometriomas and DIE compared to controls. If confirmed, these results may have important implications for prevention, clinical practice, and intervention in women with this disease.

## Figures and Tables

**Table 1:** Comparison of the general characteristics of cases of endometriosis and controls.

Characteristics	Controls (*n* = 157)	Cases (*n* = 99)	*p* *
	Mean (SD)	Mean (SD)	
Age (years)	30.7 (5.9)	36.3 (7.4)	<0.001
Height (cm)	1.64 (0.05)	1.65 (0.06)	0.88
Weight (kg)	63.2 (11.5)	63.7 (10.8)	0.74
Body mass index (kg/m^2^)	23.3 (4.3)	23.5 (3.9)	0.78
n (%)			
Diagnosed with:			
Hypertension	6 (3.8)	8 (8.0)	0.13
Diabetes	1 (0.64)	1 (1.0)	0.73
Infertility problems	13 (8.4)	28 (30.4)	<0.001
Alcohol consumption ^a^	126 (82.9)	74 (75.5)	0.15
Tobacco consumption ^b^	82 (52.9)	51 (51.5)	0.83
Educational level			
Primary	15 (9.6)	21 (21.6)	<0.001
Secondary	39 (25.0)	35 (36.1)	
University	102 (65.4)	41 (42.3)	
Marital status			
Single or divorced	77 (49.0)	32 (32.3)	0.008
Married	80 (51.0)	67 (67.7)	
Current occupation			
Unemployed	22 (14.0)	24 (24.7)	0.008
Studying	35 (22.3)	9 (9.3)	
Working	100 (63.7)	64 (66.0)	

SD: standard deviation. ^a^ Did you ever drink alcoholic beverages with a frequency of at least one a month? ^b^ Have you ever smoked? * Student’s *t* or *χ^2^* test.

**Table 2:** Comparison of the health concept scales (0–100) of the SF-12v2 questionnaire between cases and controls.

Characteristics	Controls (*n* = 157)	Cases (*n* = 99)	p *	*p* **
	Mean (SD)	Mean (SD)		
Physical Functioning	94.8 (15.5)	81.1 (29.5)	<0.001	<0.001
Physical Role	89.5 (16.5)	71.7 (28.2)	<0.001	<0.001
Bodily Pain	90.5 (18.3)	66.3 (30.7)	<0.001	<0.001
General Health	76.9 (18.9)	55.8 (26.1)	<0.001	<0.001
Vitality	65.1 (19.8)	53.1 (21.6)	<0.001	<0.001
Social Functioning	81.7 (22.4)	69.7 (24.0)	<0.001	0.001
Emotional Role	80.0 (21.1)	70.0 (25.7)	0.001	0.02
Mental Health	64.8 (18.9)	60.0 (20.5)	0.054	0.16

SD: standard deviation. * Student’s *t* test. ** Adjusted by age, educational level, marital status, infertility problems, and current occupation.

**Table 3:** Comparison of the norm-based scales and summary measure scores of SF-12v2 between cases and controls.

Characteristics	Controls (*n* = 157)	Cases (*n* = 99)	p *
	Mean (SD)	Mean (SD)	
Physical Functioning	54.7 (5.3)	50.0 (10.1)	<0.001
Physical Role	53.3 (6.1)	46.8 (10.4)	<0.001
Bodily Pain	53.6 (7.4)	43.7 (12.5)	<0.001
General Health	52.0 (8.1)	42.9 (11.2)	<0.001
PCS	56.1 (5.9)	47.7 (9.7)	<0.001
Vitality	53.8 (8.0)	49.0 (8.7)	<0.001
Social Functioning	49.2 (9.1)	44.3 (9.7)	<0.001
Emotional Role	47.2 (9.4)	42.6 (11.5)	0.001
Mental Health	47.4 (9.2)	45.0 (10.0)	0.06
MCS	46.5 (9.8)	43.8 (10.1)	0.06

* Student’s *t* test. SD: standard deviation; PCS: Physical Component Summary; MCS: Mental Component Summary.

## Data Availability

Not Applicable.
